# Aspirin induces Nrf2‐mediated transcriptional activation of haem oxygenase‐1 in protection of human melanocytes from H_2_O_2_‐induced oxidative stress

**DOI:** 10.1111/jcmm.12812

**Published:** 2016-03-10

**Authors:** Zhe Jian, Lingzhen Tang, Xiuli Yi, Bangmin Liu, Qian Zhang, Guannan Zhu, Gang Wang, Tianwen Gao, Chunying Li

**Affiliations:** ^1^ Department of DermatologyXijing HospitalFourth Military Medical UniversityXi'anShaanxiChina

**Keywords:** aspirin, melanocyte, nuclear factor E2‐related factor 2, haem oxygenase‐1, hydrogen peroxide, oxidative stress, vitiligo

## Abstract

The removal of hydrogen peroxide (H_2_O_2_) by antioxidants has been proven to be beneficial to patients with vitiligo. Aspirin (acetylsalicylic acid, ASA) has antioxidant activity and has great preventive and therapeutical effect in many oxidative stress‐relevant diseases. Whether ASA can protect human melanocytes against oxidative stress needs to be further studied. Here, we investigated the potential protective effect and mechanisms of ASA against H_2_O_2_‐induced oxidative injury in human melanocytes. Human melanocytes were pre‐treated with different concentrations of ASA, followed by exposure to 1.0 mM H_2_O_2_. Cell apoptosis, intracellular reactive oxygen species (ROS) levels were evaluated by flow cytometry, and cell viability was determined by an Cell Counting Kit‐8 assay. Total and phosphorylated NRF2 expression, NRF2 nuclear translocation and antioxidant response element (ARE) transcriptional activity were assayed with or without Nrf2‐siRNA transfection to investigate the possible molecular mechanisms. Concomitant with an increase in viability, pre‐treatment of 10‐90 μmol/l ASA resulted in decreased rate of apoptotic cells, lactate dehydrogenase release and intracellular ROS levels in primary human melanocytes. Furthermore, we found ASA dramatically induced NRF2 nuclear translocation, enhanced ARE‐luciferase activity, increased both p‐ NRF2 and total NRF2 levels, and induced the expression of haem oxygenase‐1 (HO‐1) in human melanocytes. In addition, knockdown of Nrf2 expression or pharmacological inhibition of HO‐1 abrogated the protective action of ASA on melanocytes against H_2_O_2_‐induced cytotoxicity and apoptosis. These results suggest that ASA protects human melanocytes against H_2_O_2_‐induced oxidative stress via Nrf2‐driven transcriptional activation of HO‐1.

## Introduction

Vitiligo is an acquired depigmenting disorder of great cosmetic importance characterized by loss of melanocytes in the localized epidermis. It affects approximately 0.5–1% of the world population without any racial, sexual predilection in prevalence 1. Despite continuous progress toward an elucidation of the biochemical, genetic and immunopathological pathways in vitiligo over the past decades, the precise pathogenesis remains elusive 2. Recently, epidermal oxidative stress has been documented in vitiligo patients 3. There were several lines of evidence showing impairment in the antioxidant system and reactive oxygen species (ROS)‐mediated damage in melanocytes as well as in the whole body of vitiligo patients, supporting a free‐radical‐mediated damage as an important pathogenic event in melanocyte degeneration 4, 5, 6, 7, 8, 9. Thus, inhibition of oxidative damage may represent prime targets for development of novel therapeutic agents for vitiligo.

Aspirin (acetylsalicylic acid, ASA) is the most common type of nonsteroidal anti‐inflammatory drugs (NSAIDs) and is widely used to treat inflammation and pain. Although many of the pharmacological properties of ASA and other NSAIDs are related to their inhibition of prostaglandin biosynthesis, some of their beneficial therapeutic effects are not completely understood. Recent studies have indicated that ASA has free radical scavenging property and is capable of directly protecting different cells from the deleterious effects of oxidative stress 10, 11, 12, 13. The animal and clinical research also showed that ASA has great preventive and therapeutical effect in many oxidative stress‐relevant diseases 12, 13, 14, 15, 16, 17, 18. Zailaie first showed that low‐dose ASA could protect melanocytes from vitiligo patients against oxidative stress and increase their proliferative capacities 19. Furthermore, a preliminary clinical study has found that the disease activity was suspended in all ASA‐treated vitiligo patients 20. Although Zailaie's studies are inspiring, the research has not been replicated to determine whether ASA can protect melanocytes against oxidative stress.

Although extensive studies have been carried out, the mechanism underlining ASA's antioxidative action remains unclear. Reports have claimed ASA could increase the expression of antioxidative protein haem oxygenase‐1 (HO‐1), and enhance the nitric oxide production 21, 22. Nitric oxide are well‐known stimulant of nuclear factor E2‐related factor 2 (Nrf2)‐antioxidant response element (ARE) pathway 23. Haem oxygenase‐1 is the downstream molecule of Nrf2‐ARE pathway, and degrades haem to CO, iron and biliverdin 21, 23, 24. Therefore, the antioxidative action of ASA is probably by activating of Nrf2‐ARE pathway.

Nrf2‐ARE pathway is a central part of molecular mechanisms governing the protective function of phase II detoxification and antioxidant enzymes against oxidative stress 25. Under normal circumstances, NRF2 is sequestered in the cytoplasm by a cytosolic repressor Kelch‐like ECH‐associated protein 1 (Keap1). When inducers disrupt its complex with Keap1, NRF2 translocates to the nucleus, binds to ARE and initiates the transcription of genes coding for detoxifying enzymes and cytoprotective proteins. Therefore, any compound which possesses the capacity to alter the interaction of Nrf2–Keap1 could cause the translocation of NRF2 into nuclear to exert some cytoprotective function. Our previously studies have confirmed that Nrf2‐ARE pathway plays an important role in human melanocytes against hydrogen peroxide (H_2_O_2_)‐induced oxidative injury, and its main effecter is HO‐1 26. Further research demonstrated that the activation of Nrf2‐ARE pathway was defective in melanocytes from vitiligo patients, endowing the melanocytes more vulnerable to oxidative stress 27. Thus, regulation of Nrf2‐ARE pathway may be a promising target for vitiligo treatment.

Given the above, the purpose of this study was to investigate whether ASA exerts a protective effect on human melanocytes against H_2_O_2_‐induced oxidative damage and the underlying molecular mechanism involved. Here, we show that ASA protected human melanocytes against H_2_O_2_‐induced oxidative stress through activation of Nrf2‐ARE pathway and induction of HO‐1 expression. These findings corroborate with previous studies that implicate ASA research as a novel therapeutic strategy against vitiligo.

## Materials and methods

Please see details of the following in the Supplementary Materials online.

Chemicals
Annexin V‐FITC/PI apoptosis assay
Measurement of intracellular ROS production
Lactate dehydrogenase (LDH) release assay
Transient transfection and dual luciferase reporter assay
Laser scanning confocal immunofluorescence microscopy
Western blot analysis of Nrf2 and p‐Nrf2
Transfection of short interfering RNAs (siRNAs)
Real‐time PCR analysis

### Cell culture

Normal human primary melanocytes were isolated from human forekin specimens obtained during circumcision surgery and identified by S100 and Melan A staining. Approval was obtained from the local ethics committee of Xijing Hospital, and the study was performed in strict compliance with the principles of the Declaration of Helsinki. A written consent was taken from all donors. The melanocytes were maintained in Medium 254 (Cascade Biologics/Invitrogen, Portland, OR, USA) supplemented with Human Melanocyte Growth Supplement (Cascade Biologics/Invitrogen), 5% foetal bovine serum (Invitrogen, San Diego, CA, USA), 100 U/ml penicillin and 100 U/ml streptomycin in a humid atmosphere of 5% CO_2_ at 37°C. The human epidermal melanocyte cell line PIG1 (kindly sent by Dr Caroline Le Poole, Loyola University Chicago, Maywood, IL, USA) were immortalized by retroviral introduction of the human papillomavirus type 16 E6 and E7 genes 28 and cultured in the same condition with primary melanocytes as mentioned above (Tables 1 and 2).

**Table 1:** Primer sequences of Nrf2 siRNA

Name	Primer sequences (5′–3′ orientation)
NFE2L2‐homo‐1498	Sense: GCCCAUUGAUGUUUCUGAUTT
	Antisense: AUCAGAAACAUCAAUGGGCTT
NFE2L2‐homo‐934	Sense: CCCGUUUGUAGAUGACAAUTT
	Antisense: AUUGUCAUCUACAAACGGGTT
NFE2L2‐homo‐2226	Sense: GCACCUUAUAUCUCGAAGUTT
	Antisense: ACUUCGAGAUAUAAGGUGCTT
NC‐siRNA	Sense: UUCUCCGAACGUGUCACGUdTdT
	Antisense: ACGUGACACGUUCGGAGAAdTdT

**Table 2:** Primer sequences for real‐time PCR and accession numbers

Gene name	Primer sequences (5′–3′ orientation)	Accession number
*Nrf2*	F: CTTGGCCTCAGTGATTCTGAAGTG	NM_006164
	R: CCTGAGATGGTGACAAGGGTTGTA	
*HO‐1*	F: CAGGAGCTGCTGACCCATGA	NM_002133
	R: AGCAACTGTCGCCACCAGAA	
*NQO‐1*	F: GGATTGGACCGAGCTGGAA	NM_000903
	R: AATTGCAGTGAAGATGAAGGCAAC	
*GCLC*	F: GAAGTGGATGTGGACACCAGATG	NM_001498
	R: TTGTAGTCAGGATGGTTTGCGATAA	
*GCLM*	F: GGAGTTCCCAAATCAACCCAGA	NM_002061
	R: TGCATGAGATACAGTGCATTCCAA	
*GAPDH*	F: ATGACATCAAGAAGGTGGTG	NM_008084
	R: CATACCAGGAATGAGCTTG	

F: forward; R: reverse.

### Determination of cell viability by CCK‐8 assay

Cell viability was measured using Cell Counting Kit‐8 (CCK‐8; Beyotime Institued of Biotechnology, Haimen, China) to count living cells as described previously 29. Cell Counting Kit‐8 allows convenient assays by utilizing WST‐8, which is bioreduced by cellular dehydrogenases to an orange formazan product that is soluble in tissue culture medium. The amount of formazan produced is directly proportional to the number of living cells. Briefly, primary melanocytes or PIG1 cells were seeded into 96‐well plates at an initial density of 2 × 10^4^ cells/well with a group of blank control wells (without cells) and a group of untreated control wells (cells only treatment with medium). Each incubation was performed in six separate cell culture wells. After incubation with the indicated drugs, 10 μl of kit reagent was added into 100 μl cell solution and incubated for a further 90 min. at 37°C. Cell viability was obtained by monitoring the colour change on an ELISA plate reader (Bio‐Rad, Hercules, CA, USA) setting at an absorbance reading of 450 nm.

### Statistical analysis

Data are presented as mean ± S.D. A one‐way anova was used for multiple group comparisons. These analyses were performed by GraphPad Prism (GraphPad Software 3.0; San Diego, CA, USA). Differences between groups were considered to be significant at *P* < 0.05. Each experiment was performed in triplicate and repeated at least three times.

## Results

### Aspirin attenuated H_2_O_2_‐induced cytotoxicity in human melanocytes

**Figure 1:**
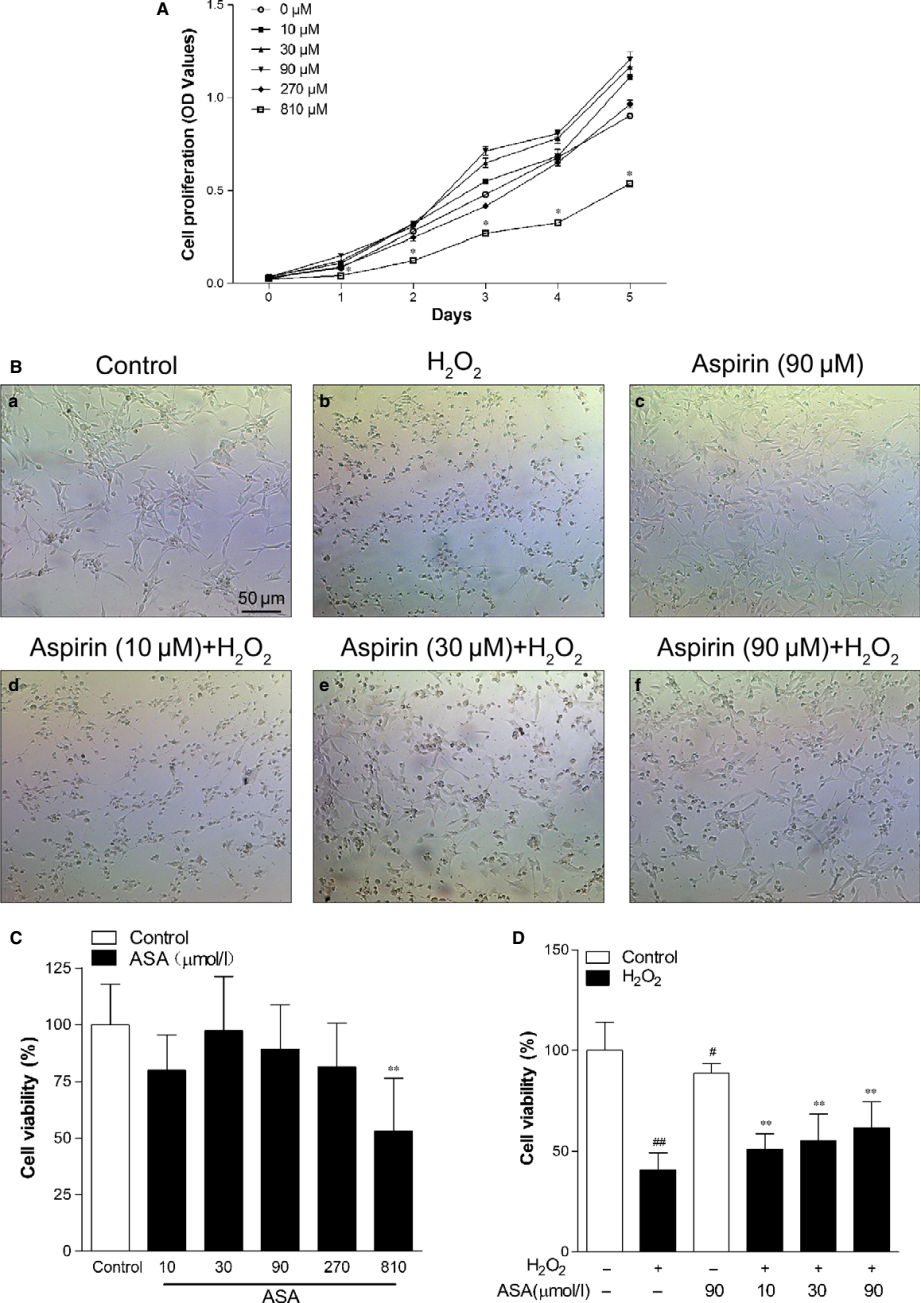
Protective effect of aspirin on H_2_O_2_‐induced cytotoxicity in primary human melanocytes. (**A**) Primary human melanocytes were treated with different concentrations of aspirin for 1–5 days, and cell proliferation was determined by CCK‐8 assay. (**B**) The effect of aspirin on H_2_O_2_‐induced morphological changes in primary human melanocytes. (**C**) Primary human melanocytes were treated with various concentrations of aspirin alone for 24 hrs, and cell viability was determined by CCK‐8 assay. (**D**) Primary human melanocytes were exposed to various concentrations of aspirin (10, 30 and 90 μM) for 24 hrs. After pre‐treatment, cells were treated with 1.0 mM H_2_O_2_ for 24 hrs, and the cell viability was determined by CCK‐8 assay. Data are presented as the mean ± S.D. of the percentages observed in treated *versus* untreated control cells from three independent experiments. *P < 0.01 *versus* untreated control cells, **P < 0.01 *versus* H_2_O_2_‐treated cells, ^#^ *P* < 0.01 *versus* H_2_O_2_‐treated cells and ^##^ *P* < 0.001 *versus* untreated control cells.

In this study, we first evaluated the effect of ASA on cell proliferation, cell viability, melanin content and tyrosinase activity of primary human melanocytes. As it can be observed in Figure 1A, melanocytes pre‐treated with ASA (10–270 μM) induced cell proliferation in a time‐dependent manner, whereas melanocytes pre‐treated with 810 μM ASA significantly inhibited cell growth compare to untreated group. However, ASA (10–810 μM) did not affect melanin content and tyrosinase activity (Fig. S1). The morphologic changes of melanocytes showed that treatment of ASA alone for 24 hrs had no significant effect on cell morphology at the concentrations ranging from 10 to 90 μM. However, 810 μM ASA resulted in cytotoxicity, including cellular dendrites shortening and partial cell death (data not shown). Although the result obtained from CCK‐8 assay demonstrated that 10–270 μM ASA alone had no significant effect on cell viability (Fig. 1C), given the results of proliferation curves and morphologic changes in melanocytes, we decided to use 10–90 μM ASA for the subsequent experiments.

Our previous work has demonstrate that treatment of melanocytes with 1.0 mM H_2_O_2_ for 24 hrs is the most appropriate way to induce consistent and high degree of oxidative damage 4, 26. Here, we investigate whether ASA protects melanocytes from H_2_O_2_‐induced cell death. Primary human melanocytes were treated with 1.0 mM H_2_O_2_ in the presence or absence of ASA (10, 30 and 90 μM), and the cell viability was assessed by cell morphology and CCK‐8 assays. After treatment of 1.0 mM H_2_O_2_ for 24 hrs, the dendrites of melanocytes shortened or disappeared (Fig. 1B, panels b) and cell viability was decreased to about 41% of the control cells (Fig. 1D). However, pre‐treatment with 10–90 μM ASA significantly attenuated H_2_O_2_‐induced oxidative damage in a dose‐dependent manner, as represented by a decreased number of injured cellular dendrites (Fig. 1B, panels e–f) and an increased cell viability of 62% great than the control cells (Fig. 1D).

### Aspirin reduced H_2_O_2_‐induced leakage of LDH and the level of intracellular ROS in human melanocytes

**Figure 2:**
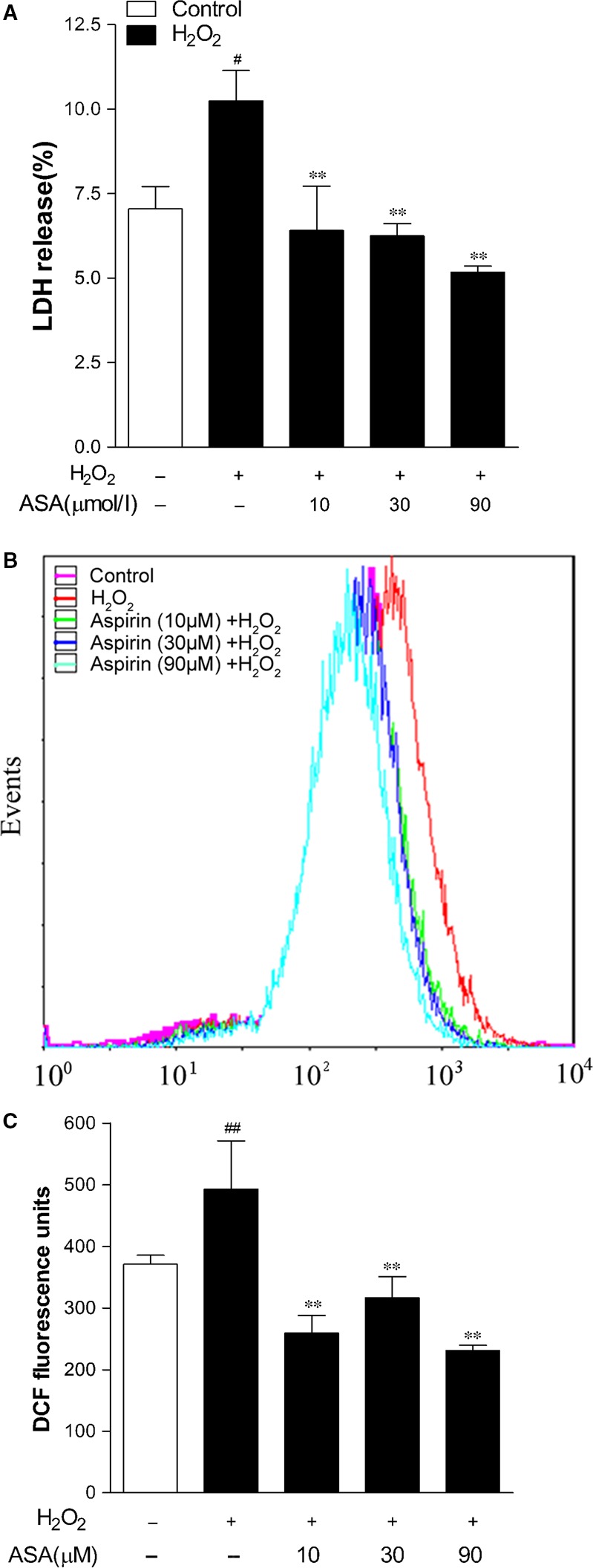
Effects of aspirin on LDH release and intracellular ROS levels in primary human melanocytes following H_2_O_2_ challenge. (**A**) LDH leakage of human melanocytes was determined by an LDH release assay. (**B**) Representative results for ROS production after pre‐treatment. (**C**) The fluorescence intensity of the cells was calculated relative to that of untreated control cells. The results shown in A and C are presented as the mean ± S.D. of three independent experiments. **P < 0.01 *versus* H_2_O_2_‐treated cells, ^#^ *P* < 0.05 and ^##^ *P* < 0.01 *versus* untreated control cells.

To further prove the protective action of ASA against oxidative damage, we determined LDH release rates and the level of intracellular ROS after treatment with 1.0 mM H_2_O_2_ for 24 hrs in primary human melanocytes. After exposure to H_2_O_2_, LDH release was significantly higher in the H_2_O_2_‐treated cells than in the control cells, indicating that H_2_O_2_ was toxic to primary human melanocytes. In accordance with CCK‐8 assay, H_2_O_2_ treatment markedly increased the LDH release rate of melanocytes and in contrast, the LDH release rate was decreased by pre‐treatment with ASA in a dose‐dependent manner (Fig. 2A).

To determine whether ASA modulates the level of ROS generated in human melanocytes in response to H_2_O_2_ treatment, we measured the intracellular level of ROS by using fluorescent probe DCFH‐DA. As shown in Figure 2B and C, treatment with H_2_O_2_ induced a robust increase in DCF fluorescence level (1.33‐fold compared to the control group). Pre‐treatment with 10–90 μM ASA for 24 hrs significantly reduced the H_2_O_2_‐induced ROS accumulation in primary human melanocytes.

### Aspirin protects human melanocytes from H_2_O_2_‐induced apoptosis

**Figure 3:**
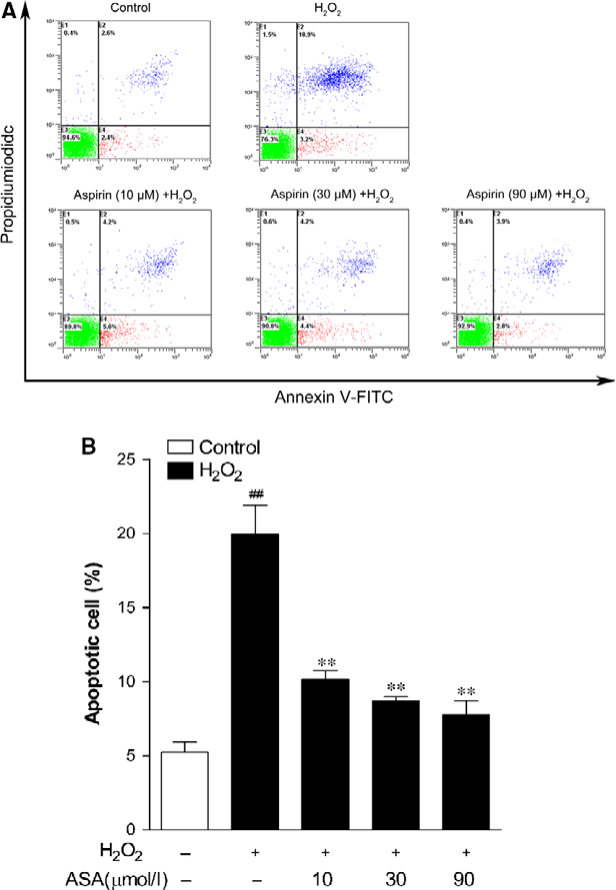
Inhibitory effect of aspirin on H_2_O_2_‐induced apoptosis in primary human melanocytes. Cells were pre‐treated with or without aspirin at the indicated concentrations for 24 hrs and then incubated in the presence or absence of 1.0 mM H_2_O_2_ for a further 24 hrs. Cellular apoptosis was assayed by annexin V‐FITC and PI counterstaining and analyzed with flow cytometry. (**A**) The original representative flow cytometry figures. (**B**) The apoptosis rates of human melanocytes. The data are presented as the mean ± S.D. of three independent experiments. **P < 0.01 *versus* H_2_O_2_‐treated cells and ^##^ *P* < 0.01 *versus* untreated control cells.

To investigate whether ASA protects against H_2_O_2_‐induced apoptosis, primary human melanocytes were pre‐treated with different concentrations of ASA (10, 30 and 90 μM) for 24 hrs before 1.0 mM H_2_O_2_ induction. To quantify the rate of cell apoptosis, flow cytometry analysis was performed with both Annexin‐V‐FITC and PI staining, the average percentages of apoptotic cells were determined as the percentage of Annexin‐V‐FITC stained cells in both Annexin‐V‐FITC and PI treated cells, the results are represented in Figure 3. Typical examples are shown in Figure 3A. After H_2_O_2_ treatment, the percentage of apoptotic cells increased to 20% from a baseline of 5.2% in the control group, whereas pre‐treatment of 10–90 μM ASA markedly inhibited H_2_O_2_‐induced apoptotic death and reduced the percentages of Annexin‐V‐stained cells up to 7.8%.

### Aspirin dramatically induces Nrf2‐mediated transcriptional activation of ARE and HO‐1, and increased NRF2 nuclear translocation, NRF2 and p‐NRF2 expression in human melanocytes

**Figure 4:**
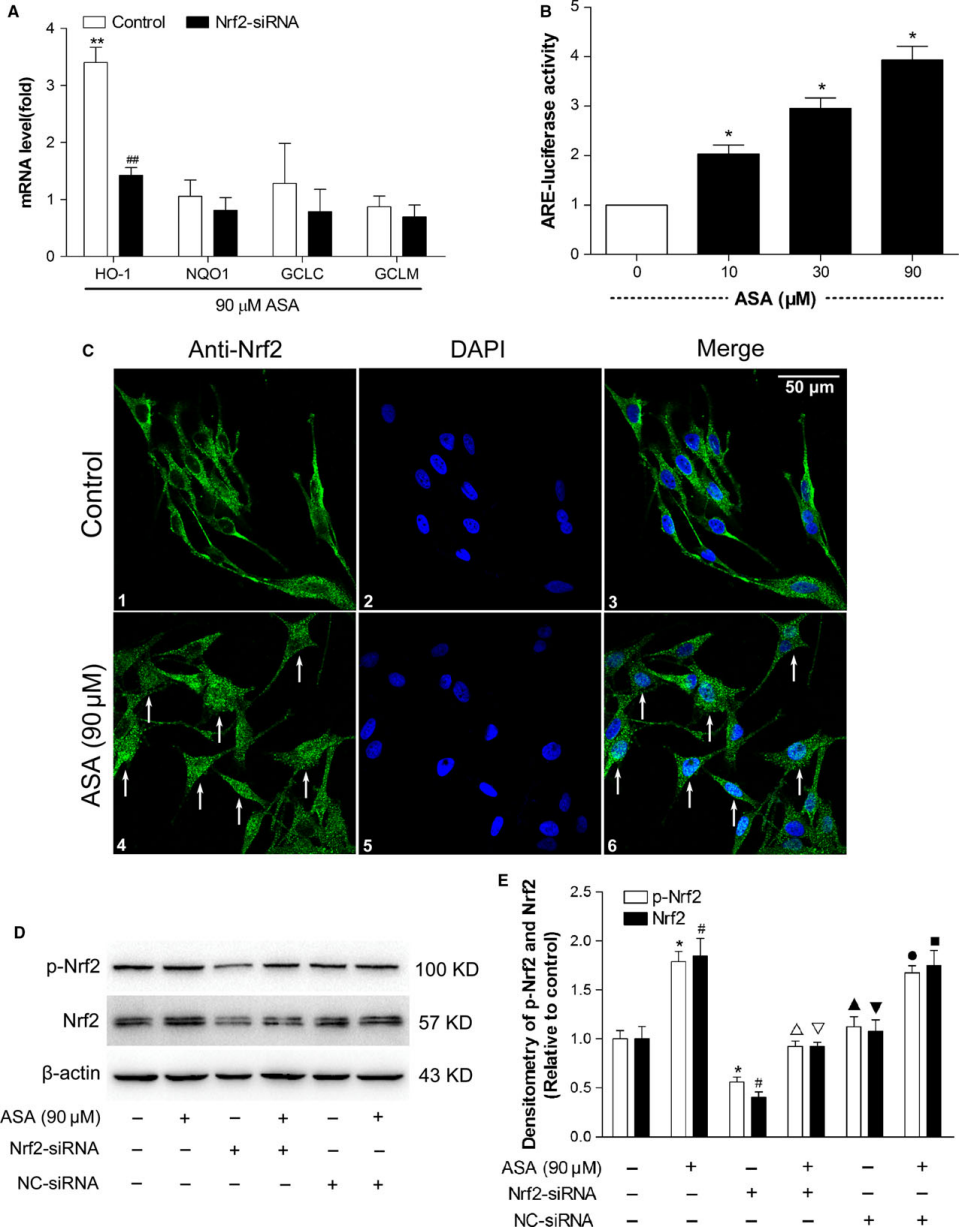
Investigation of Nrf2‐mediated phase II gene modulation, antioxidant response element (ARE) activity, Nrf2 nuclear translocation, NRF2 and p‐NRF2 protein levels in human melanocytes after treatment with aspirin. (**A**) Modulation of HO‐1, NQO‐1, GCLC and GCLM gene expression levels was detected by real‐time PCR in human melanocytes after a 24‐hr aspirin treatment with or without Nrf2‐siRNA or NC‐siRNA treatment. Data are shown as ratios of gene expression in treated cells to that in untreated controls after normalization on the basis of the expression of the GAPDH housekeeping gene. **P < 0.01 compared with the untreated cells. ^##^ *P* < 0.01 compared with the control cells. (**B**) PIG1 cells were cotransfected with an ARE‐luciferase reporter plasmid (pGL3‐ARE) and a Renilla luciferase reporter plasmid (pRLtk) for 24 hrs and treated with aspirin (10, 30 and 90 μM) for 24 hrs before luciferase activity was measured. Firefly luciferase activity in relative light units per second (RLU/s) was normalized to Renilla luciferase activity and expressed as x‐fold multiples of the control to obtain a ratio of the experimental condition to the control (control cells without ASA treatment). *P < 0.05 compared with the control cells. (**C**) NRF2 localization in melanocytes was observed by laser confocal scanning microscopy after treatment with or without aspirin for 24 hrs. Nuclear NRF2 translocation and accumulation occurred in aspirin treatment group (Arrows indicated), scale bar = 50 μm. (**D**) NRF2 and p‐NRF2 protein levels were measured by western blots after treatment with 90 μM aspirin for 24 hrs. (**E**) the intensity of each band was quantified by densitometry analysis. All protein expression was normalized to that of β‐actin, which was used as an internal control. The data are presented as the mean ± S.D. of three independent experiments. *P < 0.05 compared with p‐NRF2 in control group (untreated). ^#^ *P* < 0.05 compared with NRF2 in control group (untreated). ^▵^ *P* < 0.05 compared with p‐NRF2 in only ASA‐treated group. ^▽^ *P* < 0.05 compared with NRF2 in only ASA‐treated group. Cropped gel images are used in this figure and the gels were run under the same experimental conditions. ^▲^ *P* > 0.05 compared with p‐NRF2 in control group (untreated). ^▼^ *P* > 0.05 compared with NRF2 in control group (untreated). ^●^ *P* > 0.05 compared with p‐NRF2 in only ASA‐treated group. ^■^ *P* > 0.05 compared with NRF2 in only ASA‐treated group.

To rule out the roles of Nrf2‐driven transcriptional activation, the mRNA expression profiles of genes regulated by Nrf2 were examined by Real‐time PCR amplification from cells treated with or without ASA. In Figure 4A, treatment with ASA induced an early transcriptional activation of various Nrf2‐driven mRNAs, including HO‐1, NQO1, GCLC and GCLM. Interestingly, pre‐treatment with ASA predominantly induced much earlier and augmented expression of HO‐1 mRNA only among other Nrf2‐driven transcripts. However, transcriptional activation of other Nrf2‐driven transcripts, including as NQO1, GCLC and GCLM were not changed. To further determine whether up‐regulation of HO‐1 by aspirin treatment is caused by Nrf2 pathway, we transfected PIG1 cells with Nrf2 siRNA for 24 hrs, followed by treatment with 90 μM ASA for additional 24 hrs. As shown in Figure 4A, transfection with Nrf2 siRNA significantly reduced ASA‐mediated HO‐1 mRNA expressions compared to control.

To test our hypothesis that ASA may down‐regulate intracellular ROS and protect melanocytes from H_2_O_2_‐induced oxidative injury by activating Nrf2‐ARE pathway, we investigated the effects of ASA on ARE transcriptional activity, NRF2 nuclear translocation, p‐ NRF2 and NRF2 accumulation. As shown in Figure 4B, ASA significantly increased ARE‐luciferase activity in a dose‐dependent manner. Immunofluorescent analysis revealed that treatment of cells with 90 μM ASA for 24 hrs induced NRF2 nuclear translocation and accumulation (Fig. 4C). To further confirm whether ASA could activate NRF2 in human melanocytes, PIG1 cells were transfected with Nrf2 siRNA for 48 hrs, followed by treatment with 90 μM ASA for additional 24 hrs, and the levels of NRF2 and p‐NRF2 proteins were determined by Western blot analysis. We found that ASA treatment significantly increased the protein levels of both p‐NRF2 and NRF2, whereas transfection with Nrf2 siRNA reduced ASA‐mediated up‐regulation of p‐NRF2 and NRF2 protein expressions (Fig. 4D and E). However, cells treated with NC‐siRNA had no effect (Fig. 4D and E). These results demonstrated that ASA could induce HO‐1 expression by activating Nrf2‐ARE pathway.

### Knockdown of Nrf2 expression abrogated the protection action of Aspirin on PIG1 cells against H_2_O_2_‐induced cytotoxicity and oxidative stress

**Figure 5:**
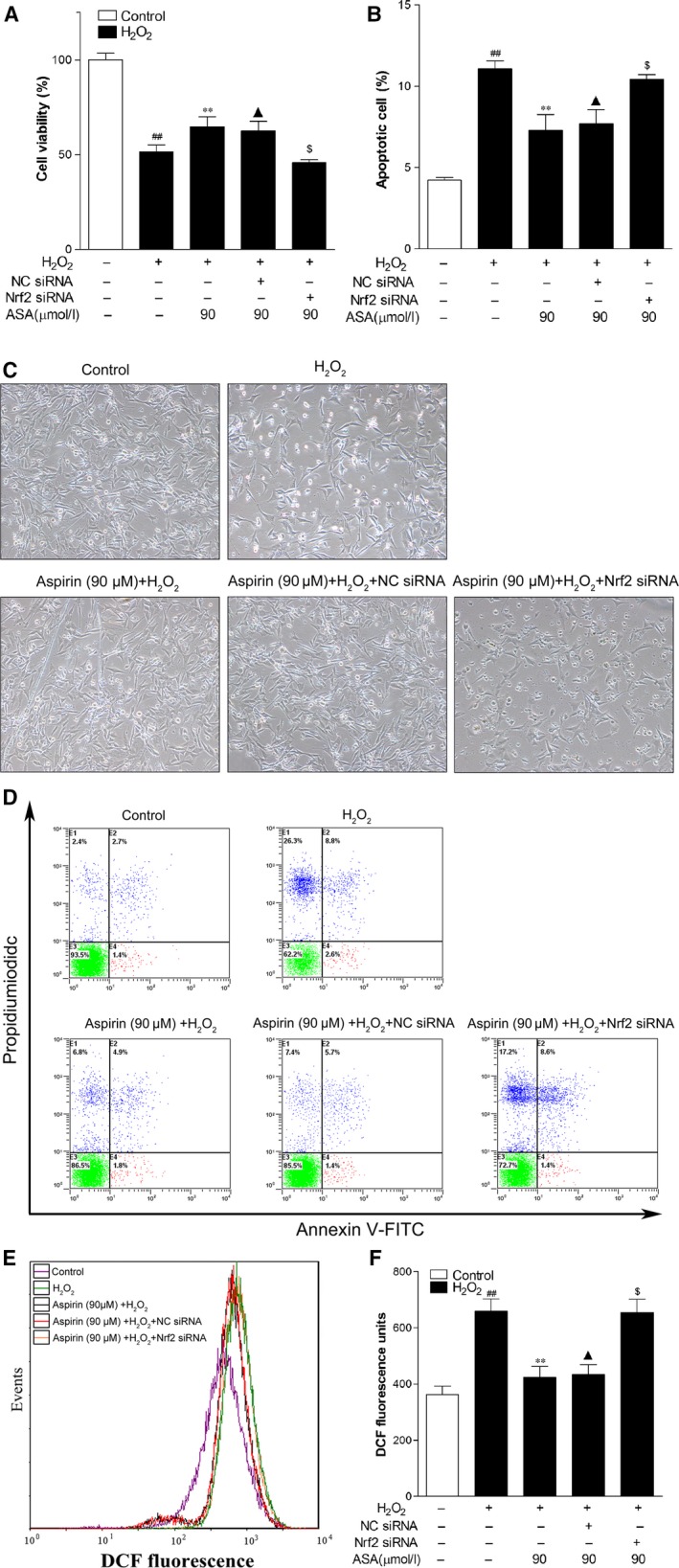
Effect of Nrf2 siRNA and aspirin on cell viability, apoptosis and intracellular ROS level induced by H_2_O_2_ in PIG1 cells. (**A**) Viability of PIG1 cells pre‐treated with Nrf2 siRNA, NC‐siRNA and/or aspirin (ASA; 90 μM) was determined with CCK‐8 assay 24 hrs after exposure to 1.0 mM H_2_O_2_ (*n* = 3). (**B**) The apoptosis rates of PIG1 cells. (**C**) The effect of Nrf2 siRNA, NC‐siRNA and aspirin on H_2_O_2_‐induced morphological changes in PIG1 cells. (**D**) The original representative flow cytometry figures. (**E**) Representative results for ROS production after pre‐treatment. (**F**) The fluorescence intensity of the cells was calculated relative to that of untreated control cells. The data are presented as the mean ± S.D. of three independent experiments. **P < 0.01 and ^$^ *P* > 0.05 *versus* H_2_O_2_‐treated cells; ^##^ *P* < 0.01 *versus* untreated control cells. ^▲^ *P* > 0.05 *versus* H_2_O_2_+ ASA treated cells.

Next, we investigated whether Nrf2‐driven transcriptional activation was directly involved in eliciting the protective effect of ASA against H_2_O_2_. PIG1 cells were transfected with specific Nrf2 ‐siRNA for 48 hrs and followed by addition of 1.0 mM H_2_O_2_ in the presence of 90 μM ASA for 24 hrs. Figure 5A and C showed that the cytoprotective effect of ASA was significantly suppressed after knockdown of Nrf2 expression, whereas transfection with NC‐siRNA had no effect. In parallel with this observation, pre‐treatment with ASA significantly decreased the H_2_O_2_‐induced apoptotic cells, which was markedly suppressed by down‐regulating the expression of Nrf2 (Fig. 5B and D). Moreover, consistent with previous results, the reduction in intracellular ROS levels by ASA in H_2_O_2_‐treated cells completely disappeared following transfection of Nrf2 siRNA (Fig. 5E and F). However, cells treated with NC‐siRNA samples did not show any change on ASA‐induced apoptosis rate and ROS level under H_2_O_2_‐treatment (Fig. 5B, D–F).

### Nrf2‐mediated HO‐1 activation is required in protective effect of Aspirin against H_2_O_2_‐induced cytotoxicity and oxidative stress

**Figure 6:**
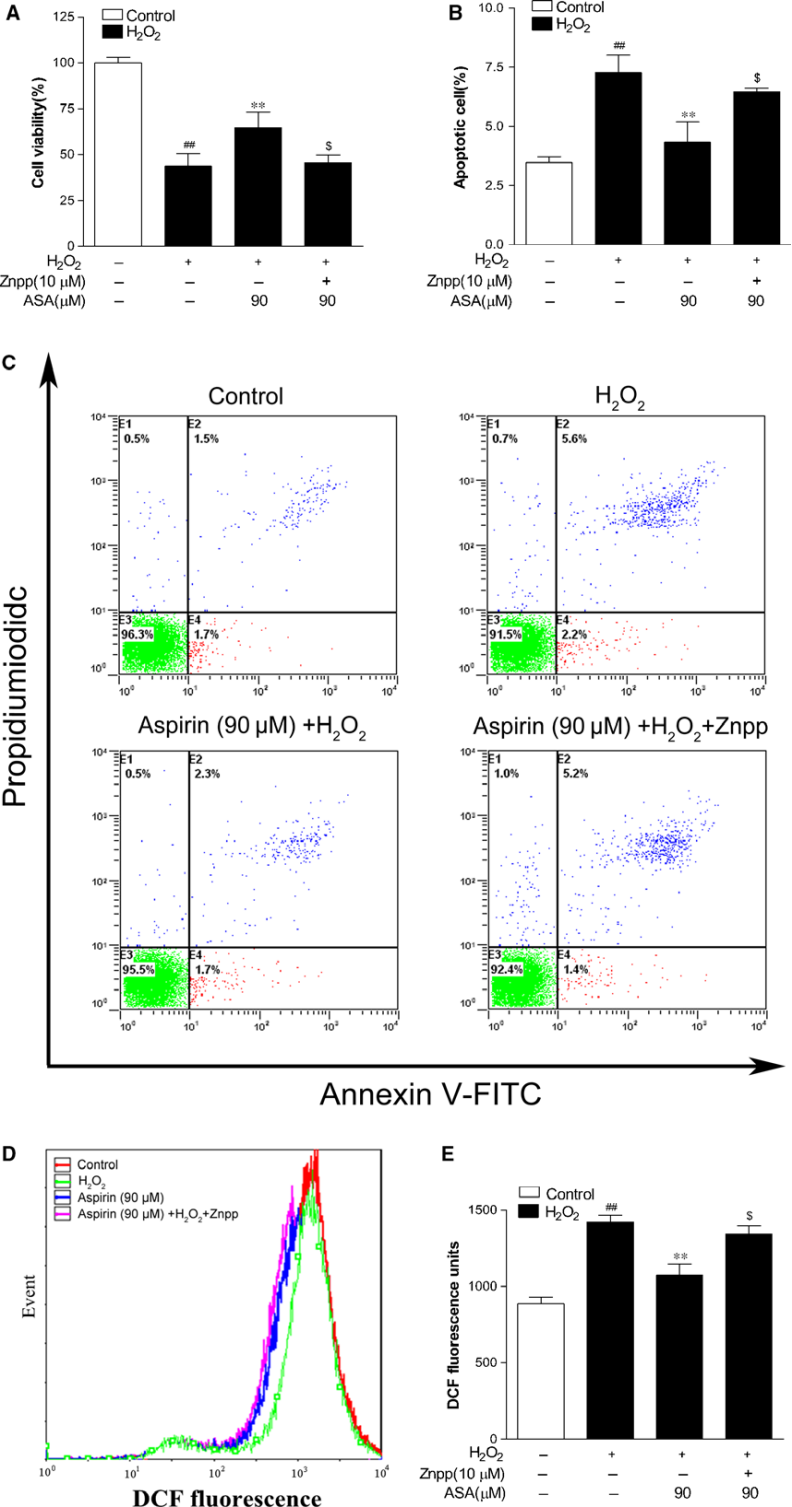
Effect of Znpp and aspirin on cell viability, apoptosis and intracellular ROS level induced by H_2_O_2_ in PIG1 cells. (**A**) Viability of PIG1 cells pre‐treated with Znpp and/or aspirin (ASA; 90 μM) was determined with CCK‐8 assay 24 hrs after exposure to 1.0 mM H_2_O_2_ (*n* = 3). (**B**) The apoptosis rates of PIG1 cells. (**C**) The original representative flow cytometry figures. (**D**) Representative results for ROS production after pre‐treatment. (**E**) The fluorescence intensity of the cells was calculated relative to that of untreated control cells. The data are presented as the mean ± S.D. of three independent experiments. **P < 0.01 and ^$^ *P* > 0.05 *versus* H_2_O_2_‐treated cells; ^##^ *P* < 0.01 *versus* untreated control cells.

To make sure that the increased expressions of HO‐1 was needed in the antioxidative property of ASA in human melanocytes against H_2_O_2_‐derived oxidative injury, we used Znpp (10 μM), the specific inhibitor of HO‐1, to pre‐treat primary human melanocytes for 24 hrs. The cells were then treated with 90 μM ASA for 24 hrs followed by 1.0 mM H_2_O_2_ for another 24 hrs. The data demonstrated that Znpp alleviated cellular protection afforded by ASA against H_2_O_2_‐mediated toxicity (Fig. 6A). We also verified that pre‐treatment with ASA resulted in a marked decrease in apoptotic cells, which was markedly suppressed by addition of Znpp (Fig. 6B and C). In addition, reduction in ROS level by pre‐treatment with ASA was also abrogated by the addition of Znpp (Fig. 6D and E).

## Discussion

Accumulating evidence indicates that oxidative stress plays a critical role in the pathogenesis of vitiligo. An extremely high level of H_2_O_2_ in lesional skin has been reported in vitiligo patients and suggested to be responsible for the disappearance of melanocytes 3. Anti‐oxidative stress‐based remedies are promising strategies for vitiligo; however, the drug development is being hampered by limited understanding of the pharmaceutically relevant molecular targets and structures in most oxidative stress‐relevant diseases.

Acetylsalicylic acid, the most widely used NSAID, had been reported to have free radical scavenging property 10, 11 and to be a potent antioxidant in many oxidative stress‐relevant diseases 12, 13, 14, 15, 16, 17, 18. Recently, Zailaie reported that ASA was potently beneficial to vitiligo patients through the inhibition of melanocytes lipid peroxidation and subsequently increased cell proliferation 19. In this study, we have further elucidated that pre‐treatment with ASA was followed by increased resistance of primary human melanocytes to oxidant injury. The capacity of ASA to protect melanocytes *in vitro* may translate into and explain the therapeutic action of ASA in vitiligo patients 19, 20. In our study, cell protection occurred at micromolar concentrations of ASA, which are well within the range of bioavailability in human oral therapy and antithrombotic dosing regimens 30, 31.

Activation of the Nrf2‐ARE signaling pathway is known to protect against oxidative stress‐induced cell death 32, 33, 34, 35, 36. Given the association of ASA with Nrf2‐ARE pathway, we explored the ASA dependent role of Nrf2‐ARE antioxidation in this study. The results demonstrated Nrf2/HO‐1 signaling directly involves in the protective effect of ASA against H_2_O_2_ in primary melanocytes. We established that HO‐1 is a key factor in ASA's protection of melanocytes against H_2_O_2_‐induced cellular damage of melanocytes. Furthermore, we proved that ASA dependent induction of HO‐1 protected melanocytes against H_2_O_2_‐induced toxicity and that inhibition of HO‐1 expression abrogated these protective effects of ASA as measured by cytotoxicity and apoptosis. Our previously published work has confirmed that Nrf2‐ARE pathway plays an important role in melanocytes against H_2_O_2_‐induced oxidative injury, and its main effecter is HO‐1 26. Further research demonstrated that the activation of Nrf2‐ARE pathway in melanocytes from vitiligo patients is defective, thus regulation of the Nrf2/HO‐1 pathway can reduced H_2_O_2_‐induced oxidative damage in human melanocytes 27. Combined with our study, we demonstrated that low dose ASA protected melanocytes from oxidative injury by activating Nrf2‐ARE pathway and inducing HO‐1 expression, which may be an approach to suspend the disease.

Although substantial progress has been made in our understanding of intracellular signaling pathways regulating Nrf2 and its target genes, further studies to clarify detailed molecular milieu of Nrf2 signaling may help design a new class of therapeutically effective antioxidants 25. Nitric oxide and its donors are well‐known antioxidant agents and stimulants of Nrf2‐ARE pathway 23, 37, 38. Acetylsalicylic acid has been reported to increase eNOS activity and enhance nitric oxide production 22, 39. These findings point to the involvement of endogenous nitric oxide as signaling molecule in ASA's antioxidative action. Whether nitric oxide is involved in the ASA‐dependent Nrf2‐ARE pathway activation need to be further investigated. Recently, a wide variety of dietary and synthetic compounds have been reported to induce Nrf2, and thereby enhancing ARE driven gene transcription to eventually exert anti‐oxidative activities 25. Our study proved ASA facilitated translocation of Nrf2 to the nucleus, where it bound to ARE, and eventually resulting in the transcriptional regulation of HO‐1 gene, which suggested it may be used as a new antioxidant. If confirmed, the cheap and most widely used NSAID, will have potent beneficial to vitiligo and other oxidative‐relevant diseases.

In conclusion, we have demonstrated that ASA protected melanocytes from oxidative injury by activation of Nrf2‐ARE pathway and induction of HO‐1 expression. Thus, ASA might represent a promising new therapeutic agent for vitiligo. More experimental and clinical research is needed to further confirm the exact effect and mechanisms of ASA in the treatment of vitiligo patients.

## Conflicts of interest

The authors declare no conflict of interest.

## Supporting information


**Figure S1** Effect of ASA on Melanogenesis in primary human melanocytes.

**Figure S2** The interference efficiency of three different Nrf2‐siRNAs.

**Data S1** Supplementary materials and methods.
