# Supplementary figures and images for: Aspirin induces Nrf2‐mediated transcriptional activation of haem oxygenase‐1 in protection of human melanocytes from H2O2‐induced oxidative stress
Source: J Cell Mol Med. 2016 Mar 10;20(7):1307–18. doi: 10.1111/jcmm.12812 (PMC4929306; doi:10.1111/jcmm.12812)

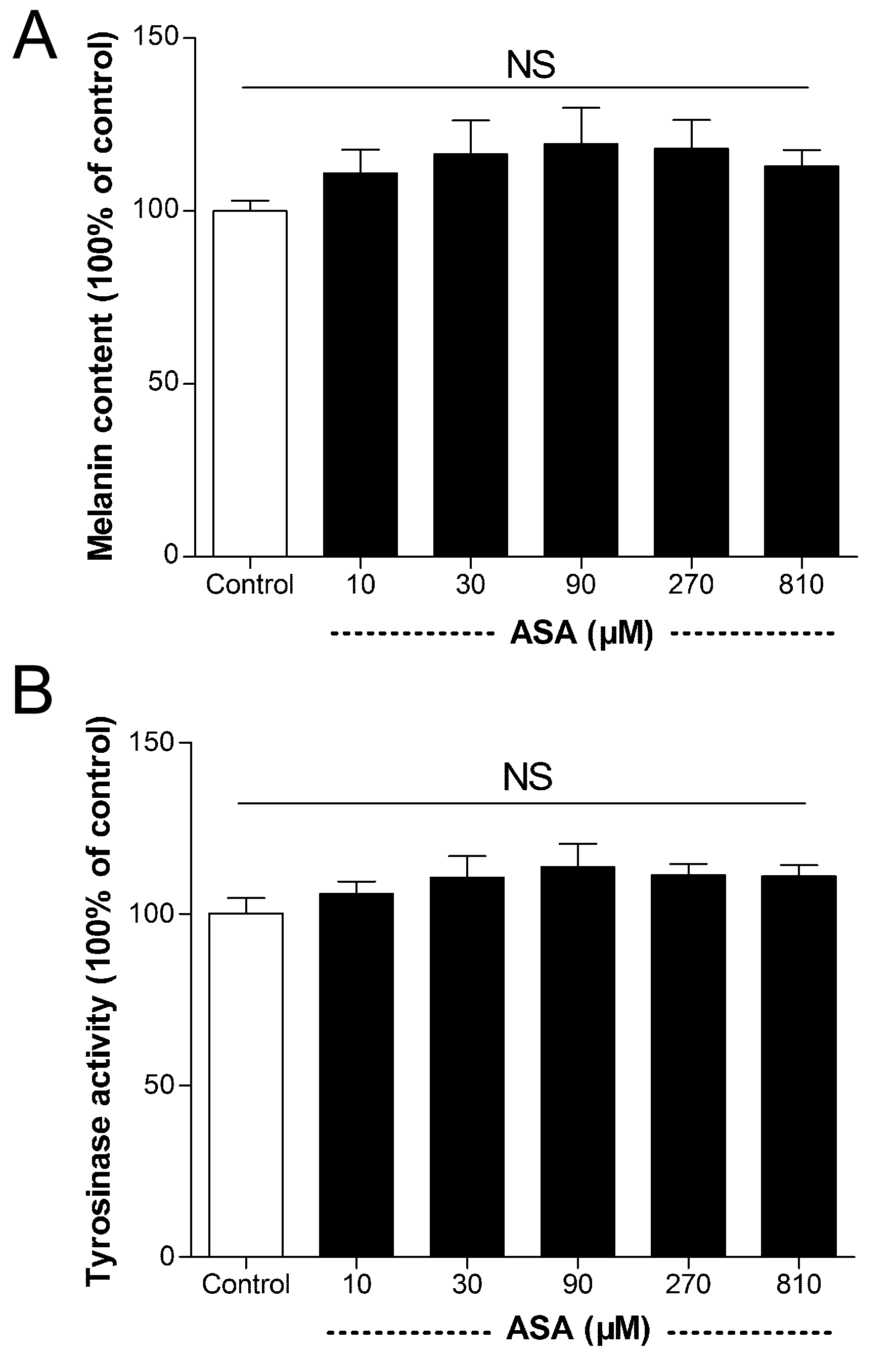

Supplement: Supplementary file 1 — Figure S1 Effect of ASA on Melanogenesis in primary human melanocytes. [file JCMM-20-1307-s001.tiff]
