# Supplementary material for: Aspirin induces Nrf2‐mediated transcriptional activation of haem oxygenase‐1 in protection of human melanocytes from H2O2‐induced oxidative stress
Source: J Cell Mol Med. 2016 Mar 10;20(7):1307–18. doi: 10.1111/jcmm.12812 (PMC4929306; doi:10.1111/jcmm.12812)
Supplement: Supplementary file 3 — Data S1 Supplementary materials and methods. [file JCMM-20-1307-s003.doc]

**Supplemental Material**

**Supplementary Materials and Methods**

**Chemicals**

The following reagents were obtained from the indicated commercial sources. Aspirin, dimethyl sulfoxide (DMSO) and ZnPP were purchased from Sigma Chemical Co. (St. Louis, MO). ZnPP were prepared in a 10 μM stock solution of 100 mM sodium hydroxide (NaOH) (Thermo-Fisher Scientific, Loughborough, UK) and were further diluted in adequate cell culture medium. Analytical pure grade H_2_O_2_ was purchased from Xi’an Chemical Reagent Factory (Xi’an, China). The concentration of H_2_O_2_ we used in the whole experiments is 1.0 mM.

**Annexin V-FITC/PI apoptosis assay**

Cell apoptosis was detected by Annexin V-FITC and propidium iodide (PI) staining according to the manufacturer’s protocol (Beijing 4A Biotech Co., Ltd, Beijing, China). In brief, primary human melanocytes or PIG1 cells were seeded into 6-well plates at the density of 5×10^6^ cells/well. Each incubation was performed in triplicate. At 24 hours after drug treatments, both floating and adherent cells were harvested and washed twice with cold phosphate-buffered saline (PBS). 5 μl Annexin V-FITC and 10 μ PI (20 μg/ml) were added to cells after resuspended in binding buffer at a density of 1×10^6^cell/ml. The mixture was incubated in the dark for 15 min at room temperature (RT). FITC and PI fluorescence was measured using flow cytometry (Beckman Coulter, Miami, FL, U.S.A.) and analyzed with Expo32 software (Beckman Coulter, Miami, FL, U.S.A.). Approximately 20,000 cells were analyzed for each sample.

**Measurement of intracellular ROS production**

Intracellular ROS production was measured with an oxidation-sensitive fluorescent probe DCF-DA (Beyotime Institued of Biotechnology, Haimen, China) as reported earlier [1]. Primary human melanocytes or PIG1 cells were seeded into 6-well plates at the density of 5×10^6^ cells/well. Each incubation was performed in triplicate. Following drug treatment, Cells were harvested by centrifugation and incubated with 10 μM DCF-DA in serum-free medium at 37 °C for 20 min. After washing twice with serum-free medium, the DCF fluorescence were immediately detected through a 525 nm band-pass filter (FL 1 channel) using flow cytometry. Approximately 20 000 cells were analyzed for each sample.

**Lactate dehydrogenase (LDH) release assay**

LDH is a soluble cytosolic enzyme present in the most eukaryotic cells. When cell membrane is damaged, it releases into the culture medium. The increase of the LDH activity in the supernatant is proportional to the number of lysed cells. Primary human melanocytes were seeded into 24-well plates at the density of 1×10^6^ cells/well. Each incubation was performed in triplicate. After indicated drugs treatment, the supernatant and cell lysates were collected, and the amount of LDH release was determined by using an LDH assay kit (Nanjing Jiancheng Bioengineering Institute, Nanjing, China) as described previously [2]. Briefly, the supernatant and cell lysates were transferred to sterile Eppendorf and incubated with 1 mg/ml NADH in pyruvate substrate solution at 37 °C for 15 min. After incubation at 37 °C for additional 15 min with 2,4-dinitrophenylhydrazine, the reaction was stopped by addition of 0.4 mM NaOH. The changes in absorbance were determined at 440 nm using a spectrophotometer (Bio-Rad, Hercules, CA, U.S.A.). LDH leakage was expressed as the percentage (%) of total LDH activity (LDH in the supernatant + LDH in the cell lysate), according to the equation: % LDH released = (LDH activity in the supernatant /total LDH activity) × 100%.

**Transient transfection and dual luciferase reporter assay**

PIG1 cells were transfected using Lipofectamine 2000 (Invitrogen, Carlsbad, CA, USA) with a pGL3-ARE reporter plasmid containing three copies of ARE (Kindly donated by Dr. Guodong Yang, Forth Military Medical University, Xi’an, China). Briefly, cells were seeded in 24-well plates at a density of 2 × 10^4^ cells/well. Cells were grown overnight, and then transfected for 6 h with 0.5 μg of pGL3-ARE plasmid, 0.05 μg of the pRLtk plasmid (Promega, Madison, WI, USA ), and the transfection reagent before being incubated for 18 h. After 24 h, the medium was replaced with fresh medium containing ASA (10, 30 and 90 μM) for 24 h. After treatment, the cells were lysed with 500 μL of passive lysis buffer (Promega). Renilla and ﬁrefly luciferase activities in cell lysates were measured with a dual luciferase assay kit (Promega) with a Glomax 20/20 luminometer (Promega). Firefly luciferase activity in relative light units per second (RLU/s) was normalized to Renilla luciferase activity and expressed as x-fold multiples of the control to obtain a ratio of the experimental condition to the control (control cells without ASA treatment).

**Laser scanning confocal immunofluorescence microscopy**

Cultured primary epidermal human melanocytes were grown as single layer on poly-l-lysine (Sigma)-coated glass slides, which were cultured in 12-well plates. After treatment with or without ASA and being washed with phosphate-buffered saline (PBS), the cells were fixed in paraformaldehyde (4%, 10 min), permeabilized with Triton X-100 (1%, 15 min), and blocked with bovine serum albumin (0.5%, 30 min) in PBS for 1 h at 37°C. Cells were then incubated with anti-Nrf2 primary antibody at a dilution of 1:50 in 1× PBS at 4°C overnight, followed by incubation with FITC-conjugated goat anti-rabbit IgG secondary antibody (Wuhan, China) at a dilution of 1:100 in 1× PBS for 30 min and nuclear dye (10 μg/ml DAPI) for 10 min at room temperature. After each step, the cells were washed 3 times with 1× PBS (0.1% Triton-100) for 10 min each. Fluorescent images were obtained by laser scanning confocal microscopy.

**Western blot analysis of Nrf2 and p-Nrf2**

After corresponding drugs treatment, cells were washed twice with cold PBS before lysed with RIPA Lysis Buffer (KeyGEN Biotech, Nanjing, China). Proteins were extracted and quantified by a BCA Protein Assay Kit (Beyotime Institued of Biotechnology, Haimen, China). Equal amounts of proteins (40 μg) were separated by 10% sodium dodecyl sulphate-polyacrylamide gel electrophoresis (SDS-PAGE, Bio-Rad) followed by transferring to a polyvinylidene difluoride membrane (Immobilon^TM^ P; Millipore, Billerica, MA, U.S.A.). The protein electrophoresis and transferring were confirmed by PageRuler Plus Prestained Protein Ladder (Fermentas, Hanover, MD, U.S.A.). After blocking in a solution of 5% non-fat dry milk diluted in Tris-buffered saline (TBS) at RT for 2 h, the membranes were incubated with primary antibody (rabbit polyclonal anti-Nrf2, 1:500, Santa Cruz Biotechnology, Santa Cruz, CA; rabbit polyclonal anti-p-Nrf2, 1:5000, Epitomics, Burlingame, California, U.S.A; mouse monoclonal anti-β-actin, 1:1000, Cwbiotec, Beijing, China) overnight at 4 °C, then the membrane were washed with TBS for 30 min and incubated with corresponding horseradish peroxidase-conjugated secondary antibodies (anti-rabbit IgG, 1:2000; anti-mouse IgG, 1:2000, Cwbiotec, Beijing, China) at RT for additional 2 h. Finally the membranes were washed with TBS for another 30 min and the membrane was incubated with ECL substrate solution for 5 min according to the manufacturer’s instructions and visualized with autoradiography film. The imaging program Quantity One (Bio-Rad) was used for quantification.

**Transfection of short interfering RNAs (siRNAs)**

Three short interfering RNAs (siRNAs) specific to the Nrf2 gene were designed and manufactured by GenePgarma (Shanghai, China). Transfection of them into PIG1 cells was carried out by using INTERFERin^TM^ (Polyplus-transfection, France) in the antibiotic-free medium according to the manufacturer’s protocol, because this transfection reagent can deliver siRNAs in the presence of serum and antibodies. Briefly, healthy PIG1 cells were seeded into the 6- and 96-well plates at the density of 1.5×10^5^ cells/well and 1×10^4^ cells/well, respectively. After reaching 50% confluence, the Nrf2 siRNAs (31 ng or 2.2 pmol per well for 6-well plates and 2.4 ng or 0.17 pmol per well for 96-well plates, the final concentration of Nrf2 siRNAs was 1 nM) were diluted with serum-free Medium 254 (200 μl per well for 6-well plates and 50 μl per well for 96-well plates) and mixed vortexly with INTERFERin^TM^ (12 μl per well for 6-well plates and 1.25 μl per well for 96-well plates). Then Nrf2 siRNA/ transfection reagent complex was incubated at RT for 10 min and gently added into the 6- and 96-well plates. For assessing mRNA and protein changes, PIG1 cells in 6-well plates were harvested after incubation at 37 °C for 48 h and 72 h, respectively, For other experiment, PIG1 cells in 6- and 96-well plates were treated with ASA and/or H_2_O_2_ 48 h after transfection. Three Nrf2-specific siRNAs were listed in Table 1. The mRNA levels of Nrf2 tested 48 h after transfection revealed that the most efficient and least cytotoxic Nrf2 siRNA was NFE2L2-homo-934, therefore, it was chosen for following study to knockdown the express of Nrf2 (Fig S2). NC-siRNA (scrambled siRNA) was used as a negative control.

**Real-time PCR analysis**

Total RNA was extracted from primary human melanocytes and PIG1 cells cultured in the 6-well plates using Total RNA Extraction Kit (AnMei Biotechnology Ltd, Xi’an, China) as per the manufacturer’s protocol. The purity and concentrations of RNA were determined by reading the absorbance at optical density 260/280 nm using a spectrophotometer. First-strand cDNAs were generated from 1μg total RNA by reverse transcription using PrimeScript® RT Reagent Kit (TaKaRa Biotechnology Co., Ltd. Dalian, China) following the manufacturer’s instructions. After cDNAs synthesis, Real-time PCR reactions were carried out with SYBR® Premix Ex Taq^TM^ II (TaKaRa Biotechnology Co., Ltd. Dalian, China) on a Chromo 4 continuous fluorescence detector with a PTC-200 DNA Engine Cycler (Bio-Rad, Hercules, CA, U.S.A.) to detect the relative mRNA levels of Nrf2, HO-1, NQO-1, GCLC, GCLM, and glyceraldehyde-3-phosphate dehydrogenase (GADPH, internal control). Reactions were run in a 20 μL volume in triplicate with 2 μL of cDNA solution and 18 μL of a homemade target-specific mix composed of 10 μL 2×SYBR® Premix Ex Taq^TM^ II and 0.5 μL 100 μM forward and 0.5 μL 100μM reverse primers solution(Augct Biotechnology, Beijing, China). All the primers used were listed in Table 2. The PCR program was as follows: 95 °C for 30 s followed by 40 cycles of denaturation at 95 °C for 5 s, annealing at 60 °C for 30 s, and extension at 72 °C for 30 s. The specificity of the PCR amplification was verified by a dissociation curve analysis. All procedures were sequenced to ensure authenticity. The genes expressions were analyzed by the 2_T_^-△△C^ (Livak) method [3].

**Supplemental Data 1. Detection of melanin content and tyrosinase activity after treatment with various concentrations of ASA for 48 h in primary human melanocytes.**

**Measurement of melanin content**

Melanin content was measured according to the method of Tsuboi et al [4] with slight modifications. Briefly, after treatment with various concentrations of ASA for 48 h melanocytes were treated with test substances for 2 days, and cell pellets containing a known number of cells (5×10^5^) were dissolved in 0.5 ml of 1 M NaOH at 100˚C for 30 min and centrifuged for 20 min at 16,000 ×g. Optical densities (OD) of supernatants were measured at 405 nm using an ELISA reader (Thermo Fisher Scientific Inc.).

**Measurement of tyrosinase activity**

Tyrosinase activity was determined as described by Tomita et al [5] with slight modification. Primary human melanocytes were plated at a density of 2.5×10^3^ cells/well in 96-well plates. After treatment with various concentrations of ASA for 48 h, melanocytes were incubated with test substances for another 48 h, and washed with ice-cold PBS and lysed with phosphate buffer (pH 6.8) containing 1% Triton-X/PBS (90 μl/well) and then frozen at -80 °C for 30 min. After thawing and mixing, 10 μl of 1% L-DOPA was added to each well. Following incubation at 37 °C for 2 h, the absorbance was measured at 475 nm using an ELISA reader (Thermo Fisher Scientific Inc., Waltham, MA USA).

**Figure S1.** **Effect of ASA on Melanogenesis in primary human melanocytes.** (A) The melanin content of primary human melanocytes was measured after 48h of ASA treatment, with or without test substances. Results are expressed as a percentage of the control. (B) Effect of ASA on L-DOPA oxidation activity of tyrosinase was measured (%). Results are expressed as a percentage of the control. The data are presented as the mean ± SD of three independent experiments.

**Supplemental Data 2: The interference efficiency of Nrf2-siRNAs.**

**Figure S2. The interference efficiency of three different Nrf2-siRNAs.** Immortalized PIG1 normal human melanocyte cell line was transfected with three different Nrf2-specific siRNAs for 48 h, and the Nrf2 mRNA expression was detected by real-time PCR analysis. The three Nrf2-siRNAs can all inhibit the expression of Nrf2, but NFE2L2-homo-934 is the most efficient one. NC-siRNA had no effect on Nrf2 mRNA expression. Data are shown as ratios of gene expression in treated cells to that in untreated control after normalization on the basis of the expression of the GAPDH housekeeping gene. **P<0.001 compared with the untreated control cells.

**References**

1 Li J, Tang Q, Li Y, *et al*. Role of oxidative stress in the apoptosis of hepatocellular carcinoma induced by combination of arsenic trioxide and ascorbic acid. *Acta Pharmacol Sin.* 2006; **27**: 1078-1084.

2 Zhang L, Yu H, Zhao X, *et al*. Neuroprotective effects of salidroside against beta-amyloid-induced oxidative stress in SH-SY5Y human neuroblastoma cells. *Neurochem Int.* 2010; **57**: 547-555.

3 Livak K, Schmittgen T. Analysis of relative gene expression data using real-time quantitative PCR and the 2(-Delta Delta C(T)) Method. *Methods.* 2001; **25**: 402-408;

4 Tsuboi T, Kondoh H, Hiratsuka J, *et al*. Enhanced melanogenesis induced by tyrosinase gene-transfer increases boron-uptake and killing effect of boron neutron capture therapy for amelanotic melanoma. *Pigment cell research.* 1998; **11**: 275-282.

5 Tomita Y, Maeda K, Tagami H. Melanocyte-stimulating properties of arachidonic acid metabolites: possible role in postinflammatory pigmentation. *Pigment cell research.* 1992; **5**: 357-361.
